# Task-relevant representations and cognitive control demands modulate functional connectivity from ventral occipito-temporal cortex during object recognition tasks

**DOI:** 10.1093/cercor/bhab401

**Published:** 2021-12-17

**Authors:** Francesca M Branzi, Clara D Martin, Pedro M Paz-Alonso

**Affiliations:** Department of Psychological Sciences, Institute of Population Health, University of Liverpool, Liverpool L69 7ZA, UK; MRC Cognition & Brain Sciences Unit, University of Cambridge, Cambridge CB2 7EF, UK; BCBL. Basque Center on Cognition, Brain and Language, San Sebastian 20009, Spain; IKERBASQUE, Basque Foundation for Science, Bilbao 48013, Spain; BCBL. Basque Center on Cognition, Brain and Language, San Sebastian 20009, Spain; IKERBASQUE, Basque Foundation for Science, Bilbao 48013, Spain

**Keywords:** cognate, fMRI, language production, semantic, ventral occipito-temporal cortex

## Abstract

The left ventral occipito-temporal cortex (vOTC) supports extraction and processing of visual features. However, it has remained unclear whether left vOTC-based functional connectivity (FC) differs according to task-relevant representations (e.g., lexical, visual) and control demands imposed by the task, even when similar visual-semantic processing is required for object identification. Here, neural responses to the same set of pictures of meaningful objects were measured, while the type of task that participants had to perform (picture naming versus size-judgment task), and the level of cognitive control required by the picture naming task (high versus low interference contexts) were manipulated. Explicit retrieval of lexical representations in the picture naming task facilitated activation of lexical/phonological representations, modulating FC between left vOTC and dorsal anterior-cingulate-cortex/pre-supplementary-motor-area. This effect was not observed in the size-judgment task, which did not require explicit word-retrieval of object names. Furthermore, retrieving the very same lexical/phonological representation in the high versus low interference contexts during picture naming increased FC between left vOTC and left caudate. These findings support the proposal that vOTC functional specialization emerges from interactions with task-relevant brain regions.

## Introduction

Imagine you are in the kitchen, looking at a cooking book. Suddenly, you are asked what you will prepare for dessert. Your intention to speak will likely drive your attention to task-relevant knowledge, in this case, the lexical and phonological representations corresponding to “cake” (e.g., Strijkers et al. 2012; Strijkers et al. 2017). Indeed, retrieving these types of representations is crucial in the context of responding to your interlocutor. However, it is likely that, later, when you are busy preparing this dessert, other aspects of knowledge related to “cake” will become more relevant than its lexical/phonological representations—for example, determining whether the batter has the right consistency and sweetness, or whether the cake will fit in the oven. The goal of the present study was to examine whether the intention to name an object and control demands experienced during speaking might shape interactions between visual areas dedicated to processing object identity (visual processing) and brain regions involved in lexical/phonological processing and control.

Visual object processing is supported by the left ventral occipitotemporal cortex (vOTC) (Kherif et al. 2011; Szwed et al. 2011; Caspers et al. 2013; Caspers et al. 2014; Lerma-Usabiaga et al. 2018). The posterior part of vOTC specifically supports processing and extraction of visual features (e.g., Lerma-Usabiaga et al. 2018). Thus, whether you need to prepare a cake or just utter its name, this part of the cortex will be recruited. However, interestingly, the “Interactive Account” of the vOTC proposes that this brain region integrates visuospatial features abstracted from sensory inputs with higher-level object associations (e.g., speech sounds, actions, and meanings), and that the specialization required by any given task emerges from interactions between the vOTC and other brain regions (Price and Devlin 2003, 2011). In other words, although some core visual-semantic features may always be activated by the presentation of a given object (e.g., a cake), different vOTC-based neural networks may be observed for different types of task-relevant representations (e.g., semantic associations, lexical representations, etc.) and control demands.

The hypothesis that vOTC-based functional connectivity (FC) is modulated by the above-mentioned factors aligns with evidence showing that left vOTC activity is permeable to top-down influences (e.g., Bar et al. 2006; Clarke et al. 2011; Twomey et al. 2011; Kay and Yeatman 2017; Oliver et al. 2017). What remains unknown is whether task-relevant representations and cognitive control demands such as, for instance, those imposed by increased lexical competition, also modulate interactions between left vOTC and other task-relevant brain regions.

The present study aims to address this issue by examining two related questions. The first question is whether the intention to name an object facilitates activation of task-relevant (i.e., lexical/phonological) representations by modulating coupling between areas that process object identity (left vOTC) and brain regions for lexical/phonological processing. The second question is whether control demands, imposed during speaking due to increased lexical competition, affect neural interactions between left vOTC and control regions. These are important questions because almost all daily tasks involve interactions with meaningful objects and require the implementation of cognitive processes that operate in a task- and context-dependent fashion.

We tested multilingual participants in a functional magnetic resonance imaging (fMRI) study, comparing FC from left vOTC in two tasks—a picture naming task and a size-judgment task. In the experiment, multilingual participants performed the picture naming task in both their first language (L1) and a much weaker, foreign language (L3), but here we are only interested in their L1 responses. Importantly, both L1 picture naming and L1 size-judgment tasks relied on similar picture processing operations (extraction of visual features, visual-semantic processing for object recognition, production of a verbal response), but only one, the picture naming task, required explicit retrieval of object names. In contrast, the L1 size-judgment task required participants to make a size-judgment providing a verbal response to indicate whether an object was “bigger” or “smaller” than an oven, but no explicit retrieval of the object name.

The first goal of the present study was to examine whether the “intention to name an object” modulated coupling between a brain region that processes object identity (left vOTC) and control regions that support lexical/phonological access. To this end, we manipulated the cognate status of the stimuli in the two tasks (picture naming and size-judgment) and examined the “cognate effect.” The cognate status of a word is determined by the extent to which it shares orthographic and phonological features with its translation equivalent in another language. Cognates are translation words that have similar orthographic–phonological forms in two languages (e.g., tomato—English, tomate—Spanish). By contrast, non-cognates are translation equivalents that share only their meaning (e.g., apple—English, manzana—Spanish). Typically, in bilingual or multilingual speakers, behavioral and neural differences between non-cognate and cognate processing are observed during picture naming and indicate lexical/phonological activity (the “cognate effect”; Costa et al. 2000; Christoffels et al. 2007; Strijkers et al. 2010). As in previous studies, here we employed the behavioral and neural cognate effect as a proxy for lexical/phonological activity, examining how it varied as a function of the intention to name an object (Strijkers et al. 2010 see below).

Some models and studies suggest that lexical/phonological representations are activated irrespective of whether the task requires explicit retrieval of an object name (“spreading activation” see Dell 1986; Caramazza 1997; Strijkers et al. 2012; see also evidence from the picture-word interference studies, i.e., Schriefers et al. 1990; Jescheniak and Schriefers 2001; de Zubicaray et al. 2002; picture-picture interference, e.g., Tipper and Driver 1988; Bles and Jansma 2008; but see other models which do not assume spreading activation in all circumstances, e.g., Levelt 1989; Levelt et al. 1999).

In other words, the very same processing stages, from visual semantic processing to lexical and phonological retrieval, would be engaged by any task requiring visual semantic processing. But, to date, no studies have assessed whether activation spreading towards the phonological processing stage can be affected by the intention to name an object. To test this hypothesis, we manipulated the “cognate status” of picture names (see above). This allowed us to examine whether behavioral and FC differences between non-cognates and cognates were present in both the L1 picture naming and L1 size-judgment tasks. Since the cognate status of a word is defined by formal overlap and is not correlated with any perceptual or conceptual variable (e.g., Costa et al. 2000, 2005; Christoffels et al. 2007; Strijkers et al. 2010; Palomar-Garcia et al. 2015), any behavioral or FC differences found between non-cognates and cognates would reflect a purely lexical/phonological effect. If this effect were found in both tasks, it would indicate that visual semantic processing automatically activates lexical/phonological representations to the same extent in both cases.

The second goal of the present study was to examine whether “contextual control demands” required during L1 speech would also modulate interactions between left vOTC and cognitive control brain regions. To address this question, we compared behavioral performance and left vOTC-based FC during the two L1 picture naming tasks, which differed only in terms of “cognitive control contexts.” Specifically, L1 picture naming in a high interference context (HIC) involved alternating single blocks of L1 and L3 picture naming. By contrast, L1 picture naming in the low interference context (LIC) involved alternating blocks of L1 picture naming and L1 size-judgment, as described above. Previous evidence has shown that HIC places greater demands on sustained control than LIC (Abutalebi et al. 2008; Branzi et al. 2014), due to increased cross-language competition during L1 word-retrieval (Abutalebi and Green 2008). This manipulation allowed us to examine how behavioral and left vOTC-based FC during L1 picture naming varied as a function of contextual control demands.

As for our first question regarding the effect of the “intention to name an object,” we had two main hypotheses. First, in line with the proposal that left vOTC operates as a part of a network for visual object recognition integrating sensory (bottom-up) information with top-down signals (Price and Devlin 2003; Devlin et al. 2006; Price and Devlin 2011), we hypothesized that this cortical region would be activated in both the L1 picture naming and size-judgment tasks. Furthermore, given the semantic nature of both tasks, left vOTC activity should couple with the activity of “semantic processing areas,” including inferior frontal gyrus (IFG), posterior middle temporal gyrus (pMTG), inferior parietal lobe (IPL), dorsal anterior cingulate cortex/pre-supplementary motor area (dACC/pre-SMA) (Badre et al. 2005; Noonan et al. 2013; Branzi et al. 2016; Jackson 2020; Sulpizio et al. 2020), and the anterior temporal lobes (ATLs) (e.g., Patterson et al. 2007; Lambon Ralph et al. 2017; Branzi, Humphreys, et al. 2020a; Jackson 2020).

Second, we hypothesized that differences in the strength of coupling between left vOTC and these regions could reflect lexical/phonological activity related to the **“**intention to name an object,” that is, when the task required explicit word-retrieval of the object’s name (L1 picture naming task). More specifically, this would predict a behavioral cognate effect and increased FC for non-cognates compared to cognates between left vOTC and the dACC/pre-SMA, a brain region previously associated with phonological control and the cognate effect (see Palomar-Garcia et al. 2015).

As for the L1 size-judgment task, it was hard to predict from the current literature whether we should expect any behavioral and/or neural cognate effects. On the one hand, according to the dynamic principle of spreading activation, we might predict that some lexical information would be activated even when there was no need to name an object (Strijkers et al. 2012). On the other hand, there is evidence that when verbalization of an object name is not required by a task (such as our L1 size-judgment task), spreading activation may be qualitatively different (e.g., Strijkers et al. 2012), and perhaps also weaker. If so, we might not detect either behavioral or neural cognate effects during the L1 size-judgment task.

As for our second question regarding the effect of “contextual control demands” during L1 speech, we had the following hypothesis: an increase in contextual control demands would impair behavioral performance and lead to stronger coupling between left vOTC and brain structures involved in sustained control of interference. A vast number of neuroimaging and patient studies has linked the left caudate to control processes in different cognitive domains (e.g., Abutalebi et al. 2000; Peterson et al. 2002; Robles et al. 2005; Crinion et al. 2006; Crone et al. 2006; Abutalebi et al. 2008; Grogan et al. 2009; Ali et al. 2010). Thus, we expected lower accuracy and stronger FC from left vOTC to the left caudate in the HIC-L1 than the LIC-L1 naming condition.

## Materials and Methods

### Participants

A total of 30 Spanish-Basque-English multilingual volunteers took part in the experiment. The data presented in this manuscript comes from the same participants that took part in Branzi, Martin, et al. (2020b). However, as explained below, the neural and behavioral data analyzed in the present study were not included in Branzi, Martin, et al. (2020b) and vice versa. Four participants were excluded from analyses due to excessive head motion during scanning (see also “Experimental tasks and procedure” and “Preprocessing”). Furthermore, fMRI data from task blocks in which participants produced more than one erroneous response were modeled separately and excluded from the main analyses. Importantly, since the present experiment conformed to an fMRI block design, with each block including only five experimental trials, this criterion ensured that only those blocks (epochs) containing at least 80% correct responses were included. Thus, three additional participants were excluded because more than 23% of epochs had more than one error. The final study sample consisted of 23 participants (mean age = 24 ± 4 years; 12 females).

Spanish was the first and dominant language (L1) of all participants, while English was a non-dominant language, acquired later in life (i.e., L3; mean age of L3 acquisition = 5 ± 3 years). All participants were right-handed and had normal or corrected-to-normal vision. No participant had a history of major medical, neurological disorders, or had received treatment for a psychiatric disorder. The study protocol was approved by the Ethics Committee of the Basque Center on Cognition, Brain and Language (BCBL), and was carried out in accordance with the Code of Ethics of the World Medical Association (Declaration of Helsinki) for experiments involving humans. Prior to their inclusion in the study, all subjects provided informed written consent. Participants received monetary compensation for their participation.

### Stimuli

Two hundred and eight line drawings of common and concrete objects, belonging to a wide range of semantic categories (e.g., animals, body parts, buildings, furniture) were selected from the International Picture Naming Project [IPNP] database (see Szekely et al. 2004). Of the selected pictures (comprising 160 experimental and 48 filler pictures, i.e., items included in the experiment solely for the purpose of reducing predictability in task sequences; see below), 50% were cognates and the remaining 50% were non-cognates. Cognate and non-cognate experimental pictures were matched for visual complexity (according to the IPNP database) [*t*(158) = 0.141, *P* = 0.888] and for lexical frequency in Spanish and English [*t*(158) = −0.689, *P* = 0.492; and *t*(158) = −0.689, *P* = 0.730, respectively]. Finally, to ensure that cognate and non-cognate stimuli were also matched in terms of mean naming latencies, we relied again on the IPNP norms (Szekely et al. 2004), especially the “Srttot” measure, which indicates mean reaction times across all valid trials in Spanish. The items selected for cognates and non-cognates did not differ in terms of mean naming latencies [*t*(158) = 0.746, *P* = 0.457].

### Experimental Tasks and Procedure

**Figure 1:**
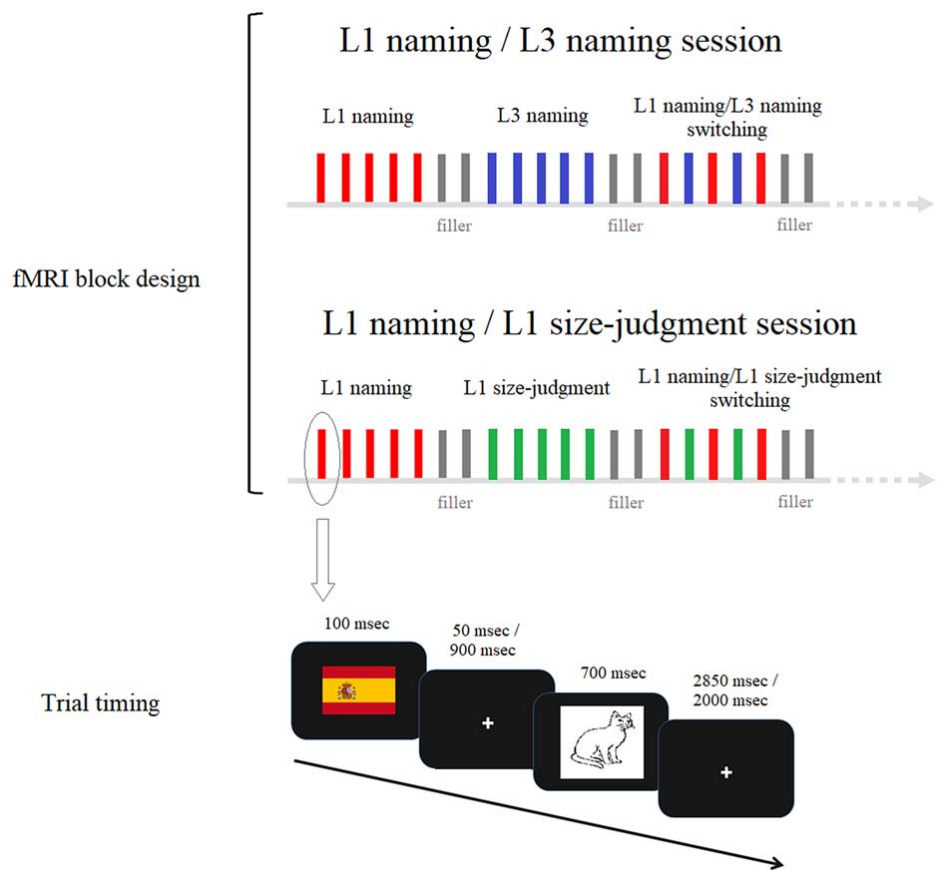
Experimental sessions and trial timing. The trials analyzed in the present study were L1 naming blocks from the L1 naming/L3 naming and the L1 naming/L1 size-judgment sessions. We also analyzed L1 size-judgment blocks from the L1 naming/L1 size-judgment session.

The experimental study included two different sessions separated by 7 ± 4 days. The order of these sessions was counterbalanced across participants (see Fig. 1). In one session (“L1 naming/L3 naming”), participants named the same pictures in both their L1 and L3 across three types of blocks: only L1 naming, only L3 naming, or continuous alternation between L1 and L3 naming trials (switching blocks). In the other session (“L1 naming/L1 size-judgment”), there were L1 picture naming blocks, L1 size-judgment blocks, and finally, blocks which required continuous alternation between L1 picture naming and the L1 size-judgment tasks (switching blocks). The L1 size-judgment task was a task requiring participants to decide whether the picture depicted an object that was “bigger” or “smaller” than an oven. Both L1 picture naming and the size-judgment task were performed using the very same pictures. As noted in the Introduction, the size-judgment task also required a verbal response for each trial: participants were instructed to utter “bigger” or “smaller,” depending on whether the object depicted in the picture was bigger or smaller than an oven.

Importantly, all tasks were identical in terms of the (I) pictures employed, (II) experimental design, and (III) timing of stimuli (see Fig. 1). Each task-session was divided into eight functional runs. Our analyses focused only on pure L1 naming and L1 size-judgment blocks. We did not include any switching blocks (i.e., blocks where either L1 and L3 naming or L1 naming and L1 size-judgment were intermixed) or any L3 naming blocks in the main analyses. Switching blocks and L3 naming blocks were modeled separately in the fMRI analyses since they were beyond the scope of the present study (see Branzi, Martin, et al. 2020b for results on switching blocks).

To address our experimental questions, we manipulated the cognate status of the pictures (cognates, non-cognates) and the type of task (HIC-L1 naming, LIC-L1 naming, and L1 size-judgment). All the task blocks included five experimental and two filler pictures. Filler pictures had the same properties as experimental pictures. However, similarly to the switching and the L3 naming blocks, they were modeled separately in the fMRI analyses. The use of filler pictures ensured that the experimental task did not favor the detection of blocks as separate entities, or any extraction of statistical regularities that could enable switch-repeat predictions.

Before participants underwent MRI scanning in each session, they received the task instructions. Then, participants were familiarized with picture names in both languages (L1/L3 naming session) or in L1 only (L1 naming/L1 size-judgment session) and performed a practice session. Instructions emphasized both speed and accuracy. During familiarization, the experimenter suggested the correct response when participants could not retrieve the name of an object depicted in a picture. This was done in order to reduce the likelihood of errors during the actual fMRI experiment. Participants were also instructed to minimize jaw–tongue movements while producing overt vocal responses to pictures, and to say “skip” when they were not able to retrieve the name of the picture.

Once inside the MRI scanner, during the “L1 naming/L3 naming” session, participants were presented with written instructions again. The first trial in each block started with a “language cue” (i.e., Spanish or English flag) presented for 100 ms, followed by the target picture, presented for 700 ms. During the time interval between the cue and the target picture (i.e., CTI), a fixation cross was presented either for 50 ms or for 900 ms. Hence, the total time between the cue and the target picture presentation was either 150 ms (i.e., short CTI) or 1000 ms (i.e., long CTI), respectively. Since every trial had a fixed duration, that is, 3 s, the time between the presentation of the target picture and the beginning of the following trial was variable (either 2850 or 2000 ms). Four resting fixation baseline intervals were included within each functional run. During this time a fixation cross was displayed for 18 s at the center of the screen. The procedure for the “L1 naming/ L1 size-judgment” session was the same as for the “L1 naming/L3 naming” session (and also included instructions and a practice session). The only difference was that participants were presented with a language-neutral “task cue,” the European flag, for the L1 size-judgment task.

In both sessions, the stimuli were presented by means of Presentation software (Neurobehavioral systems: http://www.neurobs.com/). As mentioned above, both experimental sessions used fMRI block designs which allowed us to maximize statistical power (e.g., Friston et al. 1999). Finally, vocal responses to each picture were classified as correct responses, incorrect responses, or omissions (non-responses) to assess accuracy. The background noise in the scanner did not allow us to obtain accurate measures for naming latencies. Hence, we only report the behavioral analysis for accuracy (see below).

#### Behavioral Data Analysis

For the analyses reported below, we first excluded those blocks that were not included in the fMRI analysis, that is, all the blocks in which more than one erroneous response occurred. Production of incorrect names (in naming tasks) and verbal disfluencies (stuttering, utterance repairs, and production of nonverbal sounds) were also considered erroneous responses. Conversely, for both cognates and non-cognates, responses were considered correct whenever the expected response was given, but also when participants consistently used a different but appropriate label for the target item (see the example below), as long as this did not affect its cognate status or lexical frequency. Only two participants used a non-target appropriate label, and this was for the same target item (“letterbox” instead of “mailbox”).

Behavioral analysis was performed on accuracy measures to explore different top-down modulatory effects. The effect of “intention to name an object” on lexicalization processes was assessed by comparing the cognate effect (non-cognate versus cognate) in the LIC-L1 naming versus the L1 size-judgment, using a 2 (task: LIC-L1 naming, L1 size-judgment) × 2 (cognate status: cognate, non-cognate) repeated measures analysis of variance (ANOVA). Instead, the effect of “contextual control demands” was assessed by comparing accuracy measures for HIC-L1 versus LIC-L1 naming, using a paired *t-*test.

When necessary, we applied Bonferroni correction for multiple comparisons. For the pairwise comparisons we also provided an effect size value (Cohen’s *d*) and a Bayes factor value (BF10 > 3 suggests substantial evidence for a difference between the pairs, and BF10 < 0.3 suggests substantial evidence for a null effect, see Jeffreys 1961). Reporting Bayes factors is useful for hypothesis testing because they provide a coherent approach to determining whether non-significant results support the null hypothesis over a theory, or whether the data are just insensitive. Finally, when needed, correction for non-sphericity (the Greenhouse–Geisser procedure) was applied to the degrees of freedom and *P*-values associated with factors having more than two levels.

#### MRI Data Acquisition and Analysis

Whole-brain MRI data acquisition was conducted on a 3T Siemens TRIO whole-body MRI scanner (Siemens Medical Solutions) using a 32-channel whole-head coil. Snug fitting headphones (MR Confon) were used to dampen background scanner noise and to enable communication with experimenters while in the scanner. Participants viewed stimuli back projected onto a screen by a mirror mounted on the head coil. To limit head movement, the area between participants’ heads and the coil was padded with foam; participants were asked to remain as still as possible and to minimize jaw–tongue movements while producing vocal responses. Participants’ responses were recorded with a 40 dB noise-reducing microphone system (FOMRI-III, Optoacoustics Ltd). A dual adaptive filter system subtracted the reference input (MRI noise) from the source input (naming) and filtered the production instantly while recording the output. This optic fiber microphone was also mounted on the head coil and wired to the sound filter box, whose output port was directly wired to the audio in-line plug of the computer sound card. The audio files were saved and analyzed to obtain participants’ in-scanner naming accuracy.

Functional images were acquired in eight separate runs using a gradient-echo (GE) echo-planar pulse sequence with the following acquisition parameters: time to repetition (TR) = 2500 ms, time to echo (TE) = 25 ms, 43 contiguous 3 mm^3^ axial slices, 0-mm inter-slice gap, flip angle = 90°, field of view (FoV) = 192 mm, 64 × 64 matrix, 235 volumes per run. Each functional run was preceded by four functional dummy scans to allow for T1-equilibration effects, which were later discarded. High-resolution MPRAGE T1-weighted structural images were also collected for each participant with the following parameters: TR = 2300 ms, TE = 2.97 ms; flip angle = 9°, FoV = 256 mm, voxel size = 1 mm^3^, 150 slices.

### Preprocessing

Standard SPM8 (Wellcome Department of Cognitive Neurology, London) preprocessing routines and analysis methods were employed. Images were corrected for differences in timing of slice acquisition and were realigned to the first volume by means of rigid-body motion transformation. Motion parameters extracted from the realignment were used, after a partial spatial smoothing of 4-mm full width at half-maximum (FWHM) isotropic Gaussian kernel, to inform additional motion correction algorithms implemented by the Artifact Repair toolbox (ArtRepair; Stanford Psychiatric Neuroimaging Laboratory). This allowed us to repair outlier volumes with sudden scan-to-scan motion exceeding 0.5 mm and/or 1.3% variation in global intensity via linear interpolation between the nearest nonoutlier time points (Mazaika et al. 2009).

To further limit the influence of motion on our fMRI results, we excluded participants if more than 10% of volumes in functional runs were outlier. Before applying this additional motion correction procedure, we also checked for participants who showed a drift over 3 mm/° in any of the translation (*x*, *y*, *z*) and rotation (yaw, pitch, roll) directions within each functional run. As a result of applying both of these motion correction criteria, we excluded a total of four participants from further data analyses. The average of interpolated volumes in our final sample was 2.57% (SD = 2.36%, range = 0.54–8.06%).

After volume repair, structural and functional volumes were spatially normalized to T1 and echo-planar imaging templates, respectively. The normalization algorithm used a 12-parameter affine transformation together with a nonlinear transformation involving cosine basis functions. During normalization, the volumes were sampled to 3 mm^3^ voxels. Templates were based on the MNI305 stereotaxic space (Cocosco et al. 1997), an approximation of Talairach space (Talairach et al. 1988). Functional volumes were then spatially smoothed with a 7-mm FWHM isotropic Gaussian kernel. Finally, time series were temporally filtered to eliminate contamination from slow signal drift (high-pass filter: 128 s).

### Whole-Brain Analysis

Statistical analyses were performed on individual participant data using the general linear model (GLM). The fMRI time series data were modeled by a series of impulses convolved with a canonical hemodynamic response function (HRF). The experimental conditions were modeled as 15 s epochs from the onset of the presentation of the first stimulus within each block, until the end of the presentation of the last experimental stimulus within the block. The resulting functions were used as covariates in a GLM, along with the motion parameters for translation (i.e., *x*, *y*, *z*) and rotation (i.e., yaw, pitch, roll) as covariates of noninterest. The least-squares parameter estimates of the height of the best-fitting canonical HRF for each condition were used in pairwise contrasts. Contrast images, computed on a participant-by-participant basis, were submitted to group analyses.

#### Whole-Brain Contrasts: Tasks versus Rest

According to our hypothesis, all tasks were expected to engage areas linked to object recognition and semantic cognition (e.g., Patterson et al. 2007; Lambon Ralph et al. 2017). Thus, we computed whole-brain contrasts relative to each condition of interest (HIC-L1 naming, LIC-L1 naming, and L1 size-judgment) against a passive baseline (rest) at the group level via one-sample *t* tests, treating participants as a random effect. The whole-brain statistical maps were corrected for multiple comparisons by using a voxel-level significance threshold set at *P* < 0.001, and a family wise error (FWE)-corrected cluster level significance threshold set at *P* < 0.05. Brain coordinates throughout the manuscript are reported in MNI space (Cocosco et al. 1997).

#### Whole-Brain Seed-based FC Analyses: Tasks versus Rest

The posterior part of left vOTC, corresponding to the FG2 (Caspers et al. 2013; Lorenz et al. 2017), is located posterior and dorsal to another retinotopic region phPIT (Kolster et al. 2010) in the inferior temporal gyrus. Previous studies have identified this part of the left vOTC as being responsible for visual feature extraction (e.g., Lerma-Usabiaga et al. 2018). In this study, we aimed to establish whether there were any top-down modulatory effects, driven by contextual knowledge rather than sensorial stimulation, on this process.

Thus, the anatomical left FG2 mask derived from Lorenz et al. (2017) was employed as a seed for FC analyses. It was important to use an anatomical ROI independent of our functional data to avoid potential circularity in the selection of ROIs for seed-based whole-brain FC analyses. Note that, in this study, we focused on left, rather than right, vOTC. This is because evidence suggests that language production tasks, unlike language comprehension tasks, are highly left lateralized (e.g., Gurunandan et al. 2020). Nevertheless, we also examined the data and processed the results for the right vOTC.

To identify the functional networks coupled with left vOTC during the various tasks, whole-brain seed-based FC analyses were performed implementing the beta-series correlation method (Rissman et al. 2004; Mumford et al. 2012). For this analysis, the canonical HRF in SPM was fit to each trial in each of the experimental conditions, and the resulting parameter estimates (i.e., beta values) were sorted according to task conditions to produce a condition-specific beta series for each voxel. The beta series associated with these seeds were correlated with voxels across the entire brain to produce beta correlation images for each subject for the different contrasts of interest (e.g., HIC-L1 naming > rest, LIC-L1 naming > rest, and L1 size-judgment > rest). These contrasts were subjected to an arc-hyperbolic tangent transform (Fisher 1921) to allow for statistical inference based on the magnitude of these correlations.

Group-level one-sample *t*-test FC maps were performed on the resulting subject contrast images (see above). These results were corrected for multiple comparisons by using a voxel-level significance threshold set at *P* < 0.001, and an FWE-corrected cluster level significance threshold set at *P* < 0.05. With these analyses, we were particularly interested in examining whether whole-brain FC from left posterior vOTC showed similar connectivity profiles across all tasks (HIC-L1 naming, LIC-L1 naming, and L1 size-judgment).

#### Top-Down Modulatory Effects Revealed by FC Analyses

Given our hypothesis regarding top-down modulatory effects, we determined differential coupling strength between the conditions by submitting the group maps to paired *t*-test analyses (see below). These results were corrected for multiple comparisons by applying a voxel-level significance threshold set at *P* < 0.001, and an FWE-corrected cluster level significance threshold set at *P* < 0.05. More specifically, the effect of “intention to name an object” was examined by assessing differential left vOTC whole-brain FC for non-cognates versus cognates in the LIC-L1 naming and L1 size-judgment conditions, separately. Finally, the effect of “contextual control demands” during speaking was assessed by comparing left vOTC whole-brain FC for HIC-L1 naming versus LIC-L1 naming.

## Results

### Behavioral Data

The effect of “intention to name an object” was assessed by comparing accuracy measures (% of correct responses) for LIC-L1 naming versus the L1 size-judgment, and especially the cognate effect in the two tasks. The results revealed that the main effect of “task” was not significant [*F*(1,22) = 0.184, *P* = 0.672, η*P*^2^ = 0.008, BF10 = 0.242], suggesting that the two tasks did not differ in terms of difficulty (LIC-L1 naming: Mean (*M*) = 96.903, Std. Deviation (SD) = 2.338; L1 size-judgment: *M* = 96.686, SD = 2.213). The effect of “cognate status” was also not significant [*F*(1,22) = 0.076, *P* = 0.785, η*P*^2^ = 0.003, BF10 = 0.239], (cognates: *M* = 96.739, SD = 2.09; non-cognates: *M* = 96.848, SD = 2.1). Finally, the interaction between “task” and “cognate status” was not significant [*F*(1,22) = 0.038, *P* = 0.848, η*P*^2^ = 0.002, BF10 = 0.017], (LIC-L1 naming cognates: *M* = 96.821, SD = 2.684; LIC-L1 naming non-cognates: *M* = 96.984, SD = 2.526; L1 size-judgment cognates: *M* = 96.658, SD = 2.418; L1 size-judgment non-cognates: *M* = 96.712, SD = 2.578).

The effect of “contextual control demands” during speaking was assessed by comparing accuracy measures (% of correct responses) for HIC-L1 naming (*M* = 94.905, SD = 3.133) versus LIC-L1 naming (*M* = 96.903, SD = 2.338). The results revealed a main effect of “context” [*t*(22) = −3.472, *P* = 0.002, *d* = −0.724, BF10 = 18.097], suggesting that L1 naming performance improves under low versus high interference contexts.

Overall, these behavioral results provide evidence that (1) “intention to name an object” does not modulate phonological activity (cognate effect); (2) “contextual control demands” affect L1 performance, likely due to an increase in cross-language interference for high-interference versus low-interference contexts.

### Whole-Brain Results

#### Tasks versus Rest Contrasts and FC Analyses

**Figure 2:**
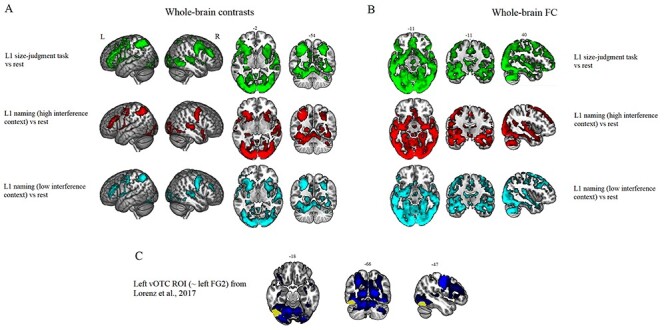
All tasks against rest. (*A*) GLM results for the different task conditions against rest. For all the contrasts, a voxel-level significance threshold was set at *P* < 0.001 with FWE correction applied at the critical cluster level set at *P* < 0.05. (*B*) FC results for each task condition against rest. These results were corrected for multiple comparisons by using a voxel-level significance threshold set at *P* < 0.001, and an FWE-corrected cluster level significance threshold set at *P* < 0.05. (*C*) The left vOTC seed (yellow) used to compute FC analyses. The seed overlaps with brain voxels commonly activated by all the tasks against rest, as revealed by the formal conjunction analysis. Conjunction analysis results were corrected for multiple comparisons by using a voxel-level significance threshold set at *P* < 0.001, and an FWE-corrected cluster level significance threshold set at *P* < 0.05.

If vOTC operates as a part of a network for visual object recognition, then this region should be positively engaged in all tasks. Indeed, we found that left vOTC was positively engaged by all tasks (see Fig. 2*A*,*C*). In line with the hypothesis that left vOTC should couple with activation in semantic areas, we found that left vOTC activation coupled with semantic regions, including IFG, pMTG, IPL, dACC/pre-SMA, and ATLs across all tasks (see Fig. 2*B*).

#### Top-Down Modulatory Effects Revealed by FC Analyses

**Figure 3:**
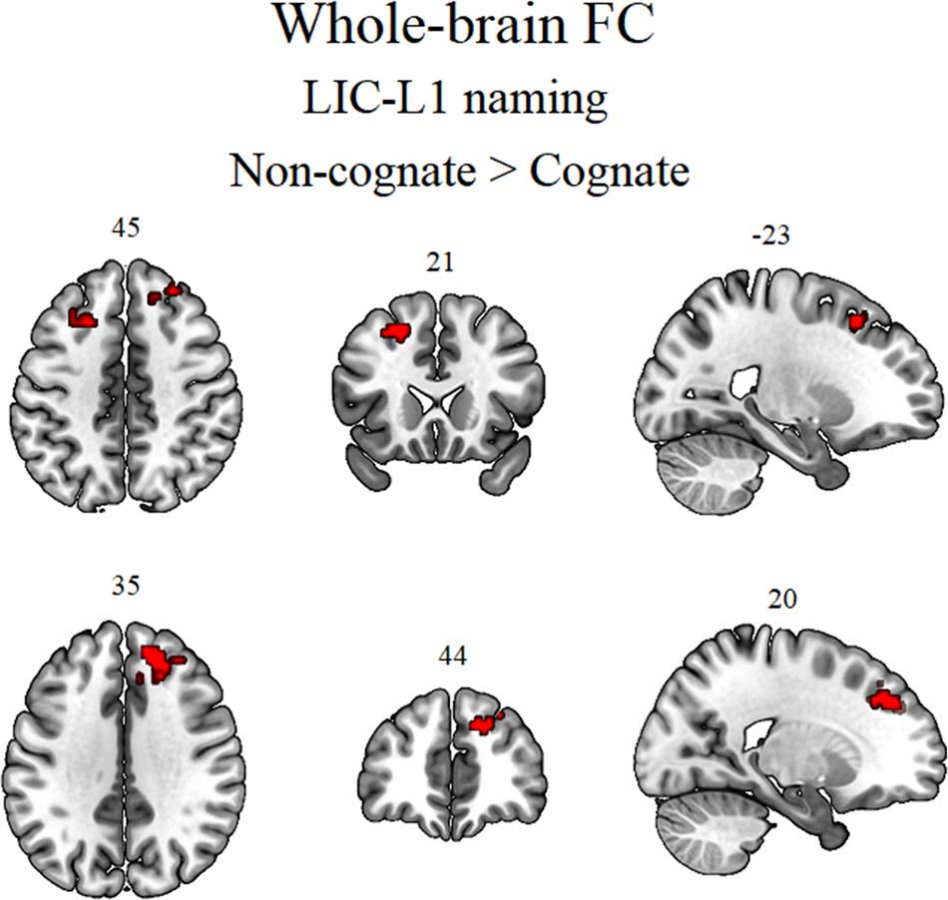
Whole-brain left vOTC-based FC results for the cognate effect (non-cognate versus cognate) in LIC-L1 naming. The results were corrected for multiple comparisons by using a voxel-level significance threshold set at *P* < 0.001, and an FWE-corrected cluster level significance threshold set at *P* < 0.05.

**Figure 4:**
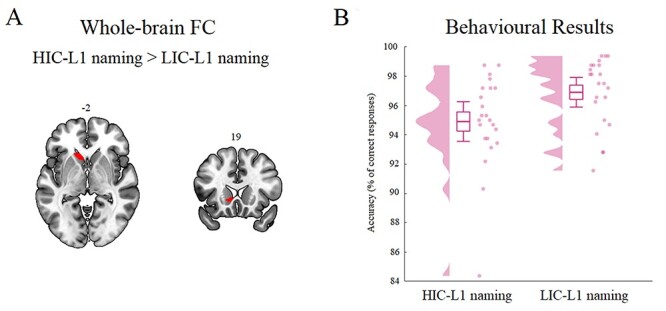
L1 naming in high versus low-interference contexts. (*A*) Left vOTC-based whole-brain FC results. The results were corrected for multiple comparisons by using a voxel-level significance threshold set at *P* < 0.001, and an FWE-corrected cluster level significance threshold set at *P* < 0.05. (*B*) Accuracy measures for HIC-L1 naming and LIC-L1 naming. The graph depicts density, the grand average (mean ± standard deviation; errors bars indicate 5th and 95th percentiles), and individual means (pink dots).

The results are summarized in Table 1 and in Figures 3 and 4. The effect of “intention to name an object” was examined by assessing the extent to which left vOTC-based FC was modulated by cognate status in L1 picture naming and the L1 size-judgment. Based on previous findings (Palomar-Garcia et al. 2015), we hypothesized that the dACC/pre-SMA would be associated with a neural cognate effect. Accordingly, during LIC-L1 naming, the cognate status (non-cognates versus cognates) of the to-be-named pictures modulated left vOTC-based FC towards right dACC/pre-SMA, extending to right dorsolateral prefrontal cortex (dlPFC) and the left frontal eye field (FEF) (see Fig. 3). By contrast, in the L1 size-judgment, cognate status did not modulate left vOTC-based FC, in line with the behavioral results.

**Table 1:** FC results (left posterior vOTC/FG2)

FG2 whole-brain FC contrast	cluster size	*T*	*x*	*y*	*z*	Location
**Intention to name an object**						
*LIC-L1 naming: non-cognates > cognates*	59	7.96	18	41	34	R Superior Frontal Gyrus
		6.07	12	47	34	R Superior Medial Gyrus
		5.29	27	38	43	R Superior Frontal Gyrus
	19	5.38	−21	20	49	L Middle Frontal Gyrus
		3.6	−30	26	46	L Middle Frontal Gyrus
**Contextual control demands**	35	4.69	−36	−94	−11	L Area hOc3v [V3v]
*HIC-L1 naming > LIC-L1 naming*		4.67	−27	−97	−14	Area hOc3v [V3v]
		4.41	−18	−94	−17	L Lingual Gyrus
	21	5.58	−12	20	−2	L Caudate Nucleus

MNI coordinates and locations of the activation peaks for FC analyses (paired *t*-tests) related to the “intention to name an object” effect, and the “contextual control demands” effect. Group level paired *t*-test FC maps were corrected for multiple comparisons using a voxel-level significance threshold set at *P* < 0.001 with FWE correction applied at the critical cluster level set at *P* < 0.05. vOTC = ventral occipital temporal cortex; L = left; R = right.

Finally, the effect of “contextual control demands” was assessed by comparing seed-based whole-brain FC for L1 naming in high versus low interference contexts (paired *t*-test). In line with the hypothesis that this contrast should induce stronger FC from left vOTC towards areas for sustained control of interference, we observed stronger FC from left vOTC towards the left caudate for HIC-L1 naming versus LIC-L1 naming (see Fig. 4). Note that, as hypothesized (see “Whole-brain seed-based FC analyses: Tasks versus rest”), FC from right vOTC did not show any significant result for the effects of “intention to name an object” and “contextual control.”

To summarize, the results (see Table 1) revealed that (1) “intention to name an object” increases FC from left vOTC to right dACC/pre-SMA reflecting phonology-related activity; and (2) “contextual control demands” during L1 naming increased FC from left vOTC to the left caudate.

## Discussion

The left posterior vOTC supports processing and extraction of visual features during object recognition, but it has remained poorly understood whether and how these processes can be influenced by top-down factors (e.g., task, context, attention, etc.). In the present fMRI study, we asked whether functional interactions between left posterior vOTC and other brain regions typically involved in semantic tasks would be affected by the intention to name an object and the control demands experienced during speaking. In line with the “Interactive Account” of the functional role of vOTC (Price and Devlin 2003, 2011), our present data identified different “vOTC networks,” including brain regions reflecting control processes during lexical and phonological access, as well as sustained control of cross-language interference, as discussed below.

### Intention to Name an Object

We asked whether the intention to name an object (explicit word-retrieval related to object recognition) proactively facilitates the activation of task-relevant representations (i.e., lexical/phonological representations), by modulating the coupling between left posterior vOTC (extraction of visual features) and brain regions involved in lexical/phonological processing. One hypothesis was that activation of visual semantic information automatically activates lexical/phonological representations (Dell 1986; Caramazza 1997; Meyer et al. 2007; Strijkers et al. 2012). If so, a non-cognate versus cognate difference in accuracy and FC measures should have been observed, irrespective of the intention to name an object, that is, in both LIC-L1 naming and L1 size-judgment tasks.

Indeed, in our study, a neural cognate effect was observed in the LIC-L1 naming, but not the L1 size-judgment task. These results revealed that intention to name an object affects lexicalization processes in a way that is not predicted by any models of language production (Dell 1986; Caramazza 1997) excepting a class of speech production models, namely “concept selection models,” which predict that only those concepts one intends to utter activate the lexicon (e.g., Levelt 1989). Our findings also align with those Stroop-like studies which reveal that distractors (i.e., words or pictures that participants do not intend to verbalize) can exert an influence on the speed of target naming, but only under certain circumstances (Jescheniak et al. 2002; Damian and Bowers 2003; Oppermann et al. 2008; Jescheniak et al. 2009; Oppermann et al. 2010).

By contrast, our results may seem at odds with previous results (Strijkers et al. 2012), which suggest that some lexical information is activated even when a task does not require explicit retrieval of the object’s name. In their study, Strijkers et al. (2012) manipulated “word frequency” and examined how the neural and behavioral “word frequency effect”—a proxy for lexical activation in that study—varied with the intention to name an object. They found a word frequency effect irrespective of the intention to speak. However, the interpretation of this result crucially hinged on the assumption that this word frequency effect reflected lexical activity. It remains unclear if this was the case, since word frequency also tends to correlate with visual and conceptual variables. Therefore, it is possible that the frequency effect observed by Strijkers et al. (2012) was not purely lexical, but rather reflected activation of a combination of visual, conceptual, and lexical information. In our study, we manipulated the cognate status of the stimuli, which is not correlated with any perceptual or conceptual variable (e.g., Costa et al. 2000, 2005; Christoffels et al. 2007; Strijkers et al. 2010; Palomar-Garcia et al. 2015). Therefore, we can confidently conclude that the neural effects observed in our study only reflect the activation of lexical/phonological representations.

Importantly, we do not argue that lexical/phonological representations are not activated in tasks that do not require explicit word-retrieval of an object name. The cognate effect refers to co-activation of both languages when a bilingual uses only one language. Thus, it remains possible that we did not observe a neural cognate effect in the L1 size-judgment because the semantic analyses required to perform this task were too superficial to engage the weak links between concepts and L3 lexical/phonological representations.

Finally, it is important to mention that the discrepancy between our and Strijkers (2012) results it is unlikely to be due to differences in the type of tasks employed. In fact, despite the semantic tasks employed in our and Strijkers (2012) studies required participants to focus on different types and number of semantic features, it is unlikely that, in our size-judgment task, any information relative to the size of the object was retrieved before participants recognized the object. Thus, as long as that task involved object identification, it is possible to evaluate the cognate effect, because spreading activation from semantic features should also reach lexical and phonological representations.

Our results provide the first evidence that a top-down intention to name an object proactively modulates the activation of lexical/phonological representations related to perceived objects, via modulation of functional interactions between left posterior vOTC and dACC/pre-SMA. Importantly, when we refer to “proactive” modulations, we mean “top-down” modulations, that is, effects that are not uniquely driven by stimulus presentation. Thus, these results indicate that left posterior vOTC and dACC/pre-SMA interactions are driven by the top-down intention to name an object, that is, are strengthened when the task at hand requires retrieving object names, particularly for non-cognate as compared to cognate stimuli.

The dACC/pre-SMA, along with the IFG, the caudate nucleus, and the left parietal cortex, is part of a set of domain-general brain regions that are also recruited during language tasks, especially when control demands increase (Duncan et al. 2020). The dACC/pre-SMA, which has been linked to the cognate effect (Palomar-Garcia et al. 2015), has also been widely associated with response monitoring processes (see Wang et al. 2007; Abutalebi and Green 2008; van Heuven et al. 2008; Abutalebi et al. 2012; Branzi et al. 2016).

With respect to the link between dACC/pre-SMA and response monitoring processes, it is important to mention that the two tasks (L1 size-judgment and L1 picture naming) might differ with respect to the number of possible responses in the response sets, and thus might entail different levels of response monitoring. However, this is unlikely to have influenced the cognate effect results. In fact, the number of possible responses for cognate and non-cognate stimuli was the same within the two tasks. Therefore, any observed difference between these two types of stimuli (i.e., any cognate effect) cannot be attributed to the number of possible responses in the response set—in accord with the lack of any significant interaction found between the factors task and cognate status.

Instead, the magnitude of the cognate effect depends only on language co-activation during a given task. Thus, our findings indicate that naming non-cognate words strengthens executive control processes related to lexical/phonological representations. Retrieval of non-cognates may be particularly challenging because speech output needs to be carefully monitored to avoid errors when competition for word selection occurs between, as well as within, languages. Cognates do not suffer from the same level of cross-language competition since there is considerable overlap between the lexical/phonological representations of both languages.

Finally, it is worth mentioning that although these results were observed using the cognate manipulation in a population of multilingual speakers, it is likely that similar findings would be observed using other types of lexical/phonological manipulations (word length, etc.) in monolingual populations. In fact, the same brain regions support word-retrieval and lexical/phonological access in monolingual and bi/multilingual speakers (e.g., Jones et al. 2012; Palomar-Garcia et al. 2015). Therefore, it is likely that an increase in lexical/phonological demands would tap into the same executive control processes and neural substrates, irrespective of the number of languages that speakers have mastered.

### Control Demands During Speaking: The Effect of Cross-language Interference

We also asked whether control demands imposed during naming could affect neural interactions between left posterior vOTC and brain regions responsible for the sustained control of interference. Our hypothesis was that an increase in control demands during speaking (due to an increase in cross-language competition) would induce strong coupling between visual areas and the left caudate. In line with this hypothesis, we found that, although L1 naming in LIC or HIC activates the same brain regions, when control demands increase so do neural interactions between left posterior vOTC and the left caudate. This shows that the brain enhances interaction between visual areas and the left caudate when it has to cope with increased control demands, and at the same time, successfully retrieve an object name.

The caudate nucleus, within the dorsal striatum, contains parallel circuits which can remove inhibition (direct pathway) or enhance inhibition (indirect and hyperdirect pathways) during task performance. Therefore, when a sensory cue indicates the need to suppress a prepotent response, projections from the cortex to dorsal striatum and vice versa are expected to control behavior, especially given changes in task rules or procedures, for example, during switching (e.g., Nambu et al. 2002; Hikosaka and Isoda 2010; Jahfari et al. 2011). This has been shown in nonverbal, but also in verbal domains. In fact, the left caudate also supports language control by keeping track of the language in use and controlling for lexical interference (Crinion et al. 2006; Ali et al. 2010; Abutalebi, Della Rosa, Ding, et al. 2013a; Abutalebi, Della Rosa, Gonzaga, et al. 2013b; Branzi et al. 2016). Our findings are consistent with the above-mentioned theories and findings. In fact, the observed increase in coupling between left posterior vOTC and the left caudate may well reflect the increased difficulty of alternating between L1 naming and L3 naming blocks, since the brain needs to engage and disengage inhibition of task-irrelevant (L3) and task-relevant (L1) representations, respectively (see Branzi et al. 2016).

Finally, previous studies have shown that the degree of left caudate involvement in language tasks depends on language proficiency. In other words, the higher the proficiency, the lower the left caudate’s involvement in the task (see Abutalebi, Della Rosa, Ding, et al. 2013a; Abutalebi, Della Rosa, Gonzaga, et al. 2013b). In line with this literature, we found stronger left caudate activity during L3 as compared to HIC-L1 naming (see Supplementary Fig. 1).

### Limitations

Due to technical issues, the behavioral results reported in this study do not include naming latencies, but only accuracy measures. Accuracy results are certainly meaningful per se and provided compelling evidence here. However, behavioral performance often involves a trade-off between accuracy and speed. Thus, to some extent, the lack of naming latencies limits our interpretation of both the neural and accuracy results.

## Conclusion

Our results provide novel evidence that task-relevant representations and cognitive control demands shape the network for visual object recognition. These findings support accounts which propose that vOTC functional specialization should emerge from regional interactions with other brain regions (Price and Devlin 2011). We identified a left posterior vOTC/dACC-pre-SMA network that may be responsible for the monitoring and phonological control, required when the presentation of a stimulus leads to concurrent activation of potentially conflicting representations. Furthermore, we revealed a cortical–subcortical network including vOTC and the left caudate that, we propose, regulates the activation of task-relevant lexical representations, possibly via sustained engagement/disengagement of inhibitory control. Future studies should investigate whether the same vOTC-networks also support monitoring and sustained control processes in tasks that do not require linguistic processing.

## Notes

The authors would like to thank Magda Altman for her useful comments on the manuscript. *Conflict of Interest:* The authors declare no competing interests.

## Funding

Francesca M. Branzi was supported by a H2020 European Research Council grant (GAP: 670428-BRAIN2 MIND_NEUROCOMP). Clara D. Martin was supported by grants from the H2020 European Research Council [ERC Consolidator Grant ERC-2018-COG-819093], the Spanish Ministry of Economy and Competitiveness [PID2020-113926GB-I00], the Basque Government [PIBA18-29]. Pedro M. Paz-Alonso was supported by grants from the Spanish Ministry of Science and Innovation [RYC-2014-15440; PGC2018-093408-B-I00], Neuroscience projects from the Fundación Tatiana Pérez de Guzmán el Bueno, and the Basque Government [PIBA-2021-1-0003]. BCBL acknowledges support by the Basque Government through the BERC 2018-2021 program and by the Spanish State Research Agency through BCBL Severo Ochoa excellence accreditation SEV-2015-0490. The authors also acknowledge the Medical Research intramural funding (MC_UU_00005/18).

## Supplementary Material

SI_Branzi_et_al_310521_bhab401
