# Supplementary material for: Task-relevant representations and cognitive control demands modulate functional connectivity from ventral occipito-temporal cortex during object recognition tasks
Source: Cereb Cortex. 2021 Dec 17;32(14):3068–80. doi: 10.1093/cercor/bhab401 (PMC9290561; doi:10.1093/cercor/bhab401)

Supplementary Information for

Task-relevant representations and cognitive control demands modulate functional connectivity from ventral occipito-temporal cortex during object recognition tasks

Francesca M. BRANZI, Clara D. MARTIN, and Pedro M. PAZ-ALONSO.

Dr Francesca M. Branzi

Email: [Francesca.Branzi@mrc-cbu.cam.ac.uk](mailto:Francesca.Branzi@mrc-cbu.cam.ac.uk)

**This Supplementary file includes:**

Figure S1

**Figure S1.** L3 naming *versus* L1 naming (high-interference context-HIC). (A) GLM results for the L3 naming *versus* L1 naming contrast in HIC. A voxel-level significance threshold was set at *p* < .001 with a FWE correction applied at the critical cluster level at *p* < .05.


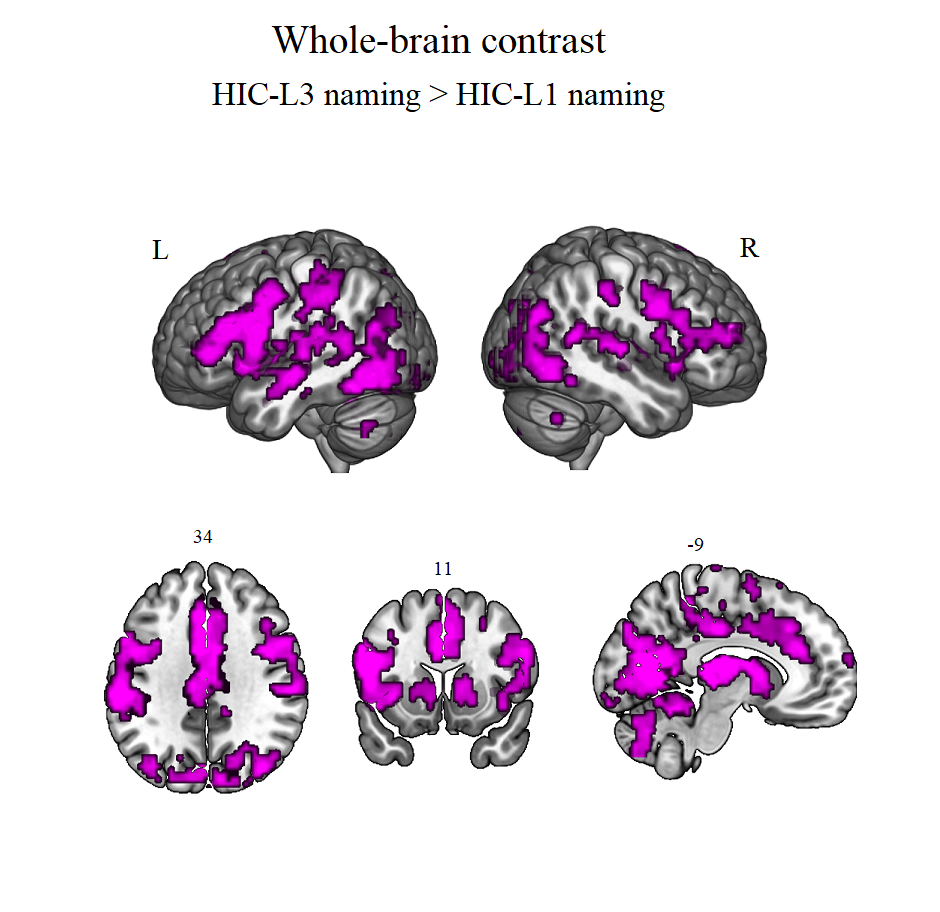

Supplement: SI_Branzi_et_al_310521_bhab401 [file si_branzi_et_al_310521_bhab401.docx]
